# LncRNA NORAD defects deteriorate the formation of age-related macular degeneration

**DOI:** 10.18632/aging.204917

**Published:** 2023-07-29

**Authors:** Jinfeng Zhang, Jing Jiang, Hongyu Zhou, Shenjun Li, Weihua Bian, Lifu Hu, Daolai Zhang, Cong Xu, Yeying Sun

**Affiliations:** 1 College of Pharmacy, Binzhou Medical University, Shandong, China; 2 Non-Clinical Research Department, RemeGen Co., Ltd, Shandong, China

**Keywords:** LncRNA NORAD, age-related macular degeneration, PGC-1α, SIRT1, senescence

## Abstract

Long noncoding RNAs (lncRNAs) play important roles in the development of age-related macular degeneration (AMD). However, the effect of long non-coding RNA activated by DNA damage (NORAD) on AMD remains unknown. This study aimed to investigate the effect of NORAD on RPE cell senescence and degeneration. Irradiated adult retinal pigment epithelial cell line-19 (ARPE-19) and sodium iodate-treated mice were used as *in vitro* and *in vivo* AMD models. Results showed that irradiation-induced AMD characteristics of ARPE-19 and NORAD-knockdown aggravated cell cycle arrest in the G2/M phase, cell apoptosis and cell senescence along with the increased expression of phosphorylated P53 (p-P53) and P21. AMD factors C3, ICAM-1, APP, APOE, and VEGF-A were also increased by NORAD-knockdown. Moreover, NORAD-knockdown increased irradiation-induced reduction of mitochondrial homeostasis factors, (i.e., TFAM and POLG) and mitochondrial respiratory chain complex genes (i.e., ND1 and ND5) along with mitochondrial reactive oxygen species (ROS). We also identified a strong interaction of NORAD and PGC-1α and sirtuin 1 (SIRT1) in ARPE-19; that is, NORAD knockdown increases the acetylation of PGC-1α. In NORAD knockout mice, NORAD-knockout accelerated the sodium iodate-reduced retinal thickness reduction, function impairment and loss of retinal pigment in the fundus. Therefore, NORAD-knockdown accelerates retinal cell senescence, apoptosis, and AMD markers via PGC-1α acetylation, mitochondrial ROS, and the p-P53–P21signaling pathway, in which NORAD-mediated effect on PGC-1α acetylation might occur through the direct interaction with PGC-1α and SIRT1.

## INTRODUCTION

Age-related macular degeneration (AMD), a multifactorial syndrome, has become the most common cause of failing vision after 70 years of age, contributes to the most prevalent single cause of blindness, and assumes increasing significance given the rising proportion of aged people in the population [1–3]. The overall incidence of AMD in the United States and Europe is estimated to exceed 30 million, and the number of patients has been growing at a rate of 5.25% per year [1]. AMD is classified as dry or wet according to clinical appearance. The most typical feature of AMD, and the preliminary focus of this work, involves the presence of drusen, which are yellow-brown spots of different sizes that deposit in the Bruch’s membrane.

AMD arises from a combination of genetic and environmental factors. The strongest environmental risk factors are age and lifestyle factors, such as smoking, oxidative stress, obesity, and diet [4, 5]. Genetic factors are known to play an important role in AMD development. The allele of the rs2230199 variant in C3 is a risk allele for AMD and a risk factor for the incidence of early AMD [6]. Complement-mediated activation of choroidal endothelial secretion of ICAM and VCAM has been hypothesized to play a role in the pathogenesis of AMD [7]. Numerous proteins, including all the major proteins of the complement pathway, namely, TIMP3, APOJ, annexin, crystallins, APOE, vitronectin, and amyloid β (Aβ), have been identified in drusen deposits [8].

Age is the most important demographic risk factor for AMD, and noncoding RNAs have a direct critical impact on the gene expression process in aging and AMD [9, 10]. Long noncoding RNAs (lncRNAs) affect the aging process by mediating various processes, including telomerase stability, intracellular communication regulation, genomic instability, epigenetic regulation, stem cell differentiation, proteostasis, and cell proliferation [11–16]. Many reports have observed changes of lncRNA expression in AMD patients, but data focused on mechanistic studies is sparse [17]. Non-coding RNA activated by DNA damage (NORAD) has recently been shown to be critical for genome stability by sequestering PUMILIO Proteins (PUM1 and PUM2) through phase separation that regulates a variety of targets in the cell, including some involved in cell growth and division [18, 19]. NORAD transcripts are located at both the cytoplasm and nucleus [18]. A total of 41 proteins, 71% in the nucleus and 5% in the cytoplasm, interacted with NORAD, including serine And Arginine Rich Splicing Factor 10 (SRSF10) and heterogeneous nuclear ribonucleoprotein (HNRNP), and they all have an RNA recognition motif found at the C-terminal of PGC-1α [20]. Florian Kopp found that NORAD-deficient drove premature aging of mice and resulted in mitochondrial dysfunction [21, 22]. However, a broad understanding of how the molecular pathways controlled by NORAD impact aging, especially aging-related diseases like AMD, remains unavailable.

Aging is associated with structural and functional changes of the retina that predispose an individual to the development of AMD and contributes to the additive effects of other pathological risk factors over time [23–25]. The role of PGC-1α in mitigating senescence has been explored that PGC-1α deficiency and inactivation of PGC-1α by acetylation induce cellular senescence with elevated mitochondrial ROS levels, abnormalities of mitochondrial structure, increased p53 levels [26, 27]. Downregulation of SIRT1 expression promotes PGC-1α acetylation and depletion of PGC-1α or SIRT1 induces senescence [26]. PGC-1α plays a pivotal role in improving mitochondrial function and ameliorating oxidative stress via the upregulation of genes for improving mitochondrial biogenesis such as the case of TFAM [28, 29]. PGC-1α has also been implicated in reactive oxygen species (ROS) detoxification and protection from neurodegenerative disease in the brain [30]. An anti-aging role of PGC-1α in a lower organism was also recently reported [31]. However, additional studies that incorporate age as a variable are needed to definitively explain the role of NORAD and PGC-1α in AMD.

Currently, several major strategies have been developed for the AMD cell model and *in vivo* model. Doxorubicin, a tumor chemotherapy agent, led to RPE senescence that was demonstrated by elevated levels of SA-β-gal staining, P53, P21, mitochondrial ROS, apoptosis, and cells in the G1 phase [32]. HiPSC-derived RPE from AMD patients can be used to model the early pathogenesis of AMD [33]. Sodium iodate (NaIO_3_), an oxidizing agent, has been extensively employed to replicate human AMD in animal models and caused thinner retinas as demonstrated by optical coherence tomography (OCT) and decreased amplitudes of the a- and b-waves as confirmed by electroretinogram (ERG) [34, 35]. A blue light exposure-induced animal AMD model showed the degeneration of the retinal layer as determined by fundus imaging [36]. However, such models remain scarce and more are needed to expand the options of researchers. The published literature has established a positive relation between X-ray lethality and age [37]. X-irradiation could result in the aging of the thoracic flight muscle of the adult housefly Musca domestic [38]. Moreover, X-irradiation-exposed HLMVEC underwent senescence (80%–85%) and cell cycle inhibition [39]. Therefore, we developed an AMD cell model which accelerated the aging of ARPE-19 cell lines by using X-irradiation.

In this study, we aimed to investigate the effects and the potential molecular mechanisms of NORAD on the AMD cell model of ARPE-19 undergoing X-ray irradiation and NaIO_3_-induced AMD in mice, thereby providing a new target for the treatment of AMD.

## RESULTS

### Irradiation induced AMD characteristics of retinal cells

**Figure 1:**
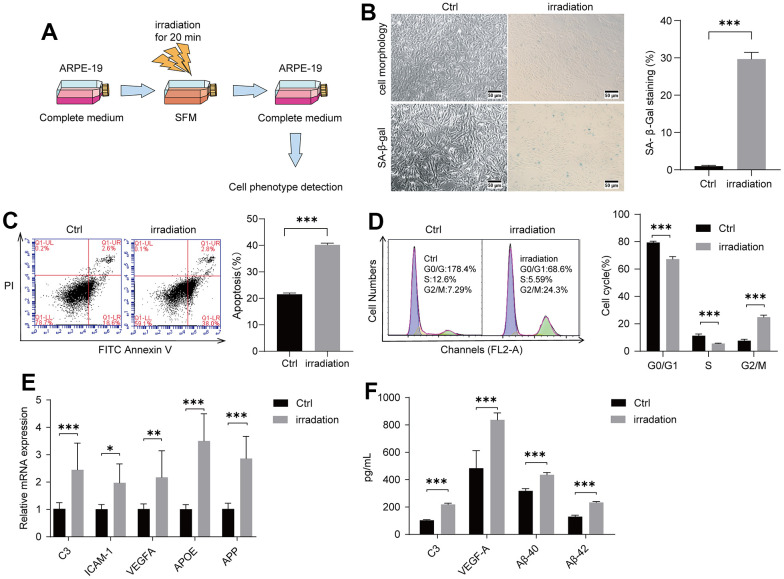
**Irradiation induced AMD markers of ARPE-19.** ARPE-19 cell lines were treated with a dose of irradiation and the cells were then observed for AMD phenotypes at different time periods. (**A**) Schematic diagram of the experimental design for AMD cell model. (**B**) Morphological observation of ARPE-19 cells at 96 h after irradiation. ARPE-19 was stained in 48 h after irradiation to determine the SA-β-gal staining. Irradiation increased the number of positive-stained cell. Results were representative of three separate experiments (100X, measure scar 50um) (**C**) Irradiation increased cell apoptosis. The apoptosis rate was detected through flow cytometry by using annexin V-FITC/PI double staining. The apoptotic rate was analyzed in 72h after irradiation in terms of the percentage of the lower and upper right quadrants. n = 3, ***P < 0.001. (**D**) Irradiation induced cell cycle arrest at G2/M phase. Flow cytometry was used to detect the cell cycle distribution of irradiation-treated ARPE-19 in 72h after irradiation. n = 3, ***P < 0.001. (**E**) RT-qPCR analysis of ARPE-19 in 96h after irradiation for AMD markers. n = 3, *P < 0.05, **P < 0.01, ***P<0.001. (**F**) ELISA testing of culture supernatant from normal ARPE-19 and irradiation-treated ARPE-19 obtained 96h after irradiation. n = 3, ***P<0.001.

Radiation exposure could damage the lens epithelial cells of the eye to induce cataracts [40, 41]. We thus examined whether irradiation could injure retinal cells to give rise to AMD. We used ARPE-19, a human retinal pigment epithelial cell line, for irradiation at 20 min under 100 cGy/min dosage rate (Figure 1A). Cell morphology changed obviously at 96 h after irradiation compared with control groups. SA-β-gal staining was also performed to assess cell senescence. The positively stained cells in the irradiated control group were obviously higher than those in the control group without irradiation (Figure 1B). Irradiation-induced apoptosis of ARPE-19 was detected via Annexin V–FITC/PI double staining. As shown in Figure 1C, irradiation increased cell apoptosis. A cell cycle experiment was then performed to examine the effect of irradiation on the ARPE-19 cell cycle progression. In irradiated cells, the percentage of ARPE-19 in the G2/M phase was enhanced compared with that in the control group, whereas the percentage of S-phase and G0/G1 cells was decreased, suggesting that the irradiation could lead to cell cycle arrest at the G2/M phase (Figure 1D). We performed a reverse transcription-quantitative polymerase chain reaction (RT-qPCR) experiment to identify that complement/inflammatory and AMD/drusen transcripts were enhanced in the irradiated group compared with the control groups (Figure 1E). In examining the secretion of C3, VEGF-A, Aβ-40, and Aβ-42 (Figure 1F) from the ARPE-19 culture supernatant medium using ELISA after irradiation, we found higher levels in the irradiated group compared with the control groups. Overall, these results suggested that irradiation could induce senescence and apoptosis of ARPE-19 cells, thereby leading to subsequent AMD formation.

### NORAD-knockdown retinal cells were more susceptible to radiation damage to form AMD

**Figure 2:**
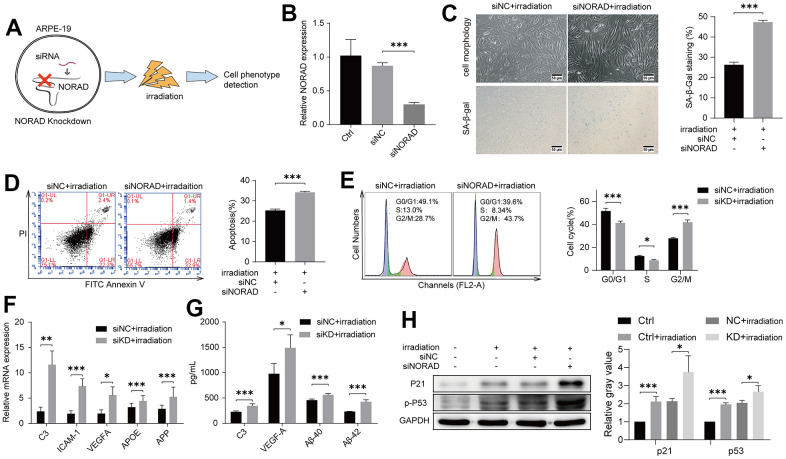
**NORAD-knockdown aggravates irradiation-induced AMD markers of ARPE-19.** ARPE-19 cell lines were transfected with siRNA of NORAD, after 24h, the cells were treated with irradiation, then observed for AMD phenotypes at different time periods. (**A**) Schematic diagram of experiment design to test the effect of NORAD on the irradiation-induced AMD model. (**B**) ARPE-19 were transfected with siNORAD or siNC. NORAD levels were analyzed through RT-qPCR. n = 3, ***P < 0.001. (**C**) ARPE-19 was observed cell morphology and stained to determine SA-β-gal. NORAD knockdown increased the number of positive-stained cells after they were treated with irradiation. Results were representative of three separate experiments. (100X, measure scar 50um) (**D**) NORAD-knockdown increased irradiation-induced cell apoptosis. The apoptosis rate was detected through flow cytometry by using annexin V-FITC/PI double staining. The apoptotic rate was analyzed in terms of the percentage of the lower and upper right quadrants. n = 3, ***P < 0.001. (**E**) NORAD silencing induced irradiation-treated cell cycle arrest at G2/M phase. Flow cytometry was used to detect the cell cycle distribution of irradiation-treated ARPE-19. n = 3, *P < 0.05, ***P < 0.001. (**F**) NORAD-knockdown increased mRNA expression of AMD markers in irradiation-treated ARPE-19 through qRT-PCR. n = 3, *P < 0.05, **P < 0.01, ***P < 0.001. (**G**) NORAD knockdown increased AMD markers of culture supernatant from irradiation-treated ARPE-19. n = 3, *P<0.05, ***P<0.001. (**H**) NORAD-knockdown increased the p-P53 and P21 expression observed through western blot. The ratio of p-P53 and P21 to GAPDH was analyzed with ImageJ. Values were shown as mean ± SD, n = 3, *P<0.05, ***P<0.001.

We next explored whether NORAD knockdown in the ARPE-19 cell line leads to the eventual worsening of AMD development after irradiation at a certain incubation time (Figure 2A). Small interfering RNA-targeting NORAD (siNORAD) was transfected into ARPE-19 to knock down the NORAD expression. The NORAD knockdown was verified and assessed through RT-qPCR. The NORAD expression in siNORAD cells was knocked down by 70% compared with the counterpart without small interfering RNA (siNC) cells (Figure 2B). Subsequently, we investigated whether NORAD knockdown increased the sensitivity of ARPE-19 to irradiation stress. Figure 2C shows that the cell morphology of the irradiated siNORAD group was significantly changed compared with that of the irradiated siNC group. In irradiation treatment cells, the percentage of SA-β-gal-positive cells in the siNORAD group was obviously enhanced compared with that in the siNC group (Figure 2C). The effect of NORAD-knockdown on the irradiation-induced apoptosis of ARPE-19 was detected via Annexin V–FITC/PI double staining. As shown in Figure 2D, NORAD knockdown intensively increased irradiation-induced cell apoptosis. In irradiated cells, the percentage of ARPE-19 in the G0/G1 and S phase was decreased in the siNORAD group compared with that in the siNC group, but the percentage of G2/M phase cells was enhanced significantly, indicating a G2/M phase arrest (Figure 2E). Then, we examined the mRNA level of AMD-related markers including complement/inflammatory and AMD/drusen transcripts using RT-qPCR and verified that the outcome for the irradiated siNORAD group was higher than that for the irradiated siNC group (Figure 2F). In analyzing the protein level of AMD-related markers ARPE-19 secreted, we used ELISA and found that NORAD-knockdown significantly accelerated the levels of C3, VEGF-A, Aβ-40, and Aβ-42 in culture supernatants (Figure 2G). Correspondingly, the levels of the aging-related proteins, p-P53 and P21, were significantly increased in irradiation-induced ARPE-19 cell lines. Surprisingly, NORAD knockdown accelerated the expression of p-P53 and P21 (Figure 2H). Taken together, these findings indicate that the irradiated siNORAD group presented more severe G2/M arrest, senescence, and apoptosis of ARPE-19 than the irradiated siNC group, thereby NORAD-knockdown in ARPE-19 promoted the sensitivity of ARPE-19 to irradiation damage.

### The retinas of NORAD knockout mice were more susceptible to sodium iodate damage

**Figure 3:**
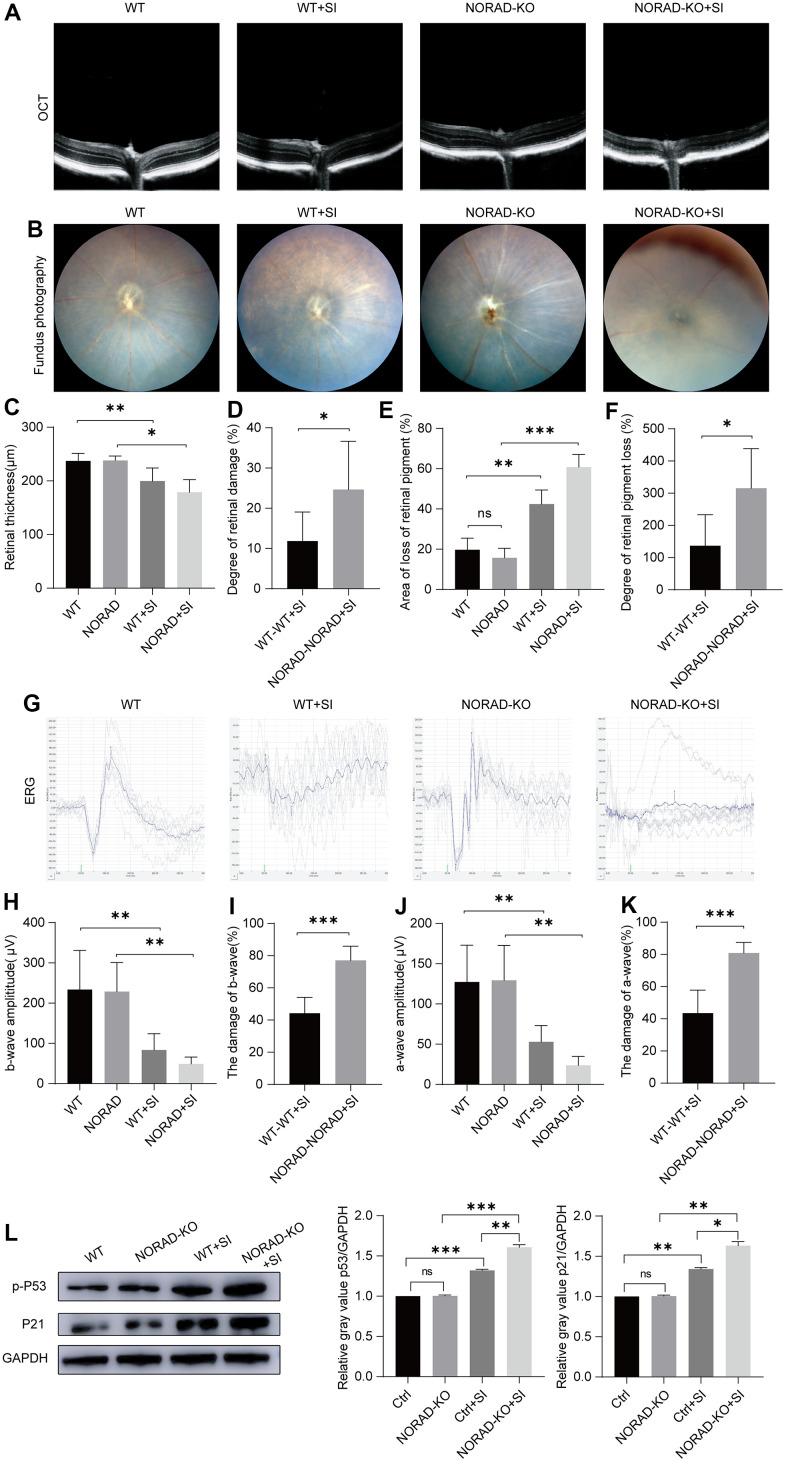
**Injury effects of NORAD knockout on the retina of NaIO_3_-treated mice.** Different techniques were used to detect retinal damage in wild-type mice and NORAD knockout mice treated with sodium iodate. (**A**) Optical coherence tomography (OCT) was performed seven days after NaIO_3_ treatment for all three study groups. (**B**) Funduscopic examinations were performed seven days after SI injection. Representative OCT and fundus fluorescence shows the RPE of the mice in each experimental group. (**C**) Statistical graph of retinal thickness. (**D**) Statistical graph of retinal damage between WT mouse and NORAD-KO mouse after SI injection. (**E**) Statistical graph of retinal pigment loss area. (**F**) Statistical graph of retinal pigment loss degree between WT mouse and NORAD-knockout mouse after SI injection. (**G**) Electroretinography was performed to investigate the function of the retina in response to light. (**H**) Statistical graph of b wave amplitude change. (**I**) Statistical graph of the degree of change in b wave amplitude between WT mouse and NORAD-knockout mouse in eight days after SI injection. (**J**) Statistical graph of a wave amplitude change. (**K**) Statistical graph of the degree of change in a wave amplitude between WT mouse and NORAD-knockout mouse in eight days after SI injection. n≥3, *P < 0.05, **P < 0.01, ***P < 0.001. (**L**) Western blot was used to detect the p-P53 and P21 expression levels. n = 3, *P < 0.05, **P < 0.01, ***P < 0.001.

To prove that NORAD-knockout could accelerate the outside damage to the retina *in vivo*, we used NORAD-knockout mice injected with NaIO_3_ in the tail vein. After 7 days of NaIO_3_ treatment, OCT was employed to observe the thickness of the entire retina (Figure 3A). Little difference was observed in retinal thickness between NORAD- knockout mice and normal wild mice (Figure 3C). When we injected the mice with NaIO_3_, retinal thickness decreased in both wild and NORAD-knockout mice (Figure 3C), but the thickness of the retinal damage in NORAD-knockout mice was greater than that in WT mice (Figure 3D). To validate the results, we performed a funduscopic examination to evaluate the retinal pigment loss (Figure 3B). Before the NaIO_3_ injection, WT and NORAD-knockout mice had similar retinas (Figure 3E). After the NaIO_3_ injection, the fundus images of WT mice showed yellow spots on the retina, representing RPE loss by NaIO_3_. By contrast, the RPE loss area of NORAD-knockout mice was beyond the WT mice with NaIO_3_ (Figure 3F). Next, we assessed the functional integrity of the retina using electroretinography (Figure 3G). The amplitudes of the a- and b-waves of WT and NORAD-knockout mice are similar before the NaIO_3_ injection (Figures 3H, 3J). Moreover, the amplitudes of the a- and b-waves decreased in NaIO_3_-injected WT mice compared with those of the WT group without NaIO_3_, thereby demonstrating functional damage of the photoreceptors and dysfunction in the bipolar cells of NaIO_3_-injected WT mice. The amplitudes of the a- and b-waves of NORAD-out mice also decreased more than that of the wild mice (Figures 3I, 3K). We then measured the expression of p-P53 and P21 proteins in the eyes of the mice. No significant change in p53 and p21 between WT mice and NORAD-knockout mice. Conversely, the increase in p-P53 and P21 in NORAD-knockout mice was greater than that in wild mice under sodium iodate injection induced AMD model. (Figure 3L). These findings indicate that the retinas of NORAD-knockout mice were more vulnerable to injury than those of WT mice, an outcome that was consistent with *in vitro* results.

### NORAD knockdown increased mitochondrial ROS production and complement 3 expressions in the eyes of mice

**Figure 4:**
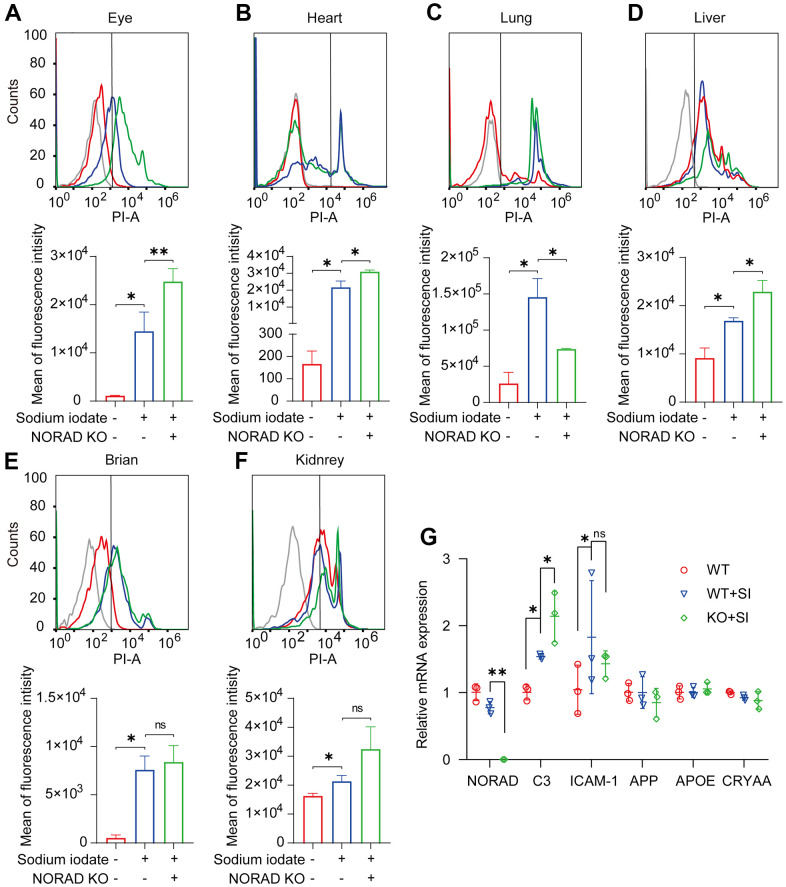
**NORAD-knockout aggravates SI-induced mitochondrial ROS and C3 expression level *in vivo*.** Mitochondrial ROS and AMD markers were detected in wild-type mice and NORAD knockout mice treated with sodium iodate. (**A**–**F**) Flow cytometry was used to detect the mitochondrial ROS levels in the eyes, heart, lungs, liver, brain, and kidneys of mice on day 8 after SI injection. n=3, *P < 0.05, **P < 0.01. (**G**) mRNA expression of AMD markers in eyes of the mouse through qRT-PCR. n = 3, *P < 0.05, **P < 0.01.

Considering that mitochondrial ROS elevation is a reason for the increase of p-P53 and P21, we tested the mitochondrial ROS production in multiple organs of mice. One week after NaIO_3_ injection, the mitochondrial ROS in the eyes, heart, liver, lung, brain, and kidney of wild mice increased significantly, and the expression of mitochondrial ROS in the eyes, heart, and liver of NORAD-knockout mice was higher than that of wild mice (Figure 4A–4F). Next, we detected AMD markers in the eyes of mice and found that the mRNA levels of C3 and ICAM-1 were significantly increased in the eyes one week after NaIO_3_ injection, and the expression of C3 was increased in NORAD-knockout mice (Figure 4G). These results suggest that NORAD-knockout exacerbates the increase of mitochondrial ROS and C3 in the eye.

### NORAD knockdown increased mitochondrial damage and mitochondrial ROS and PGC-1α acetylation

**Figure 5:**
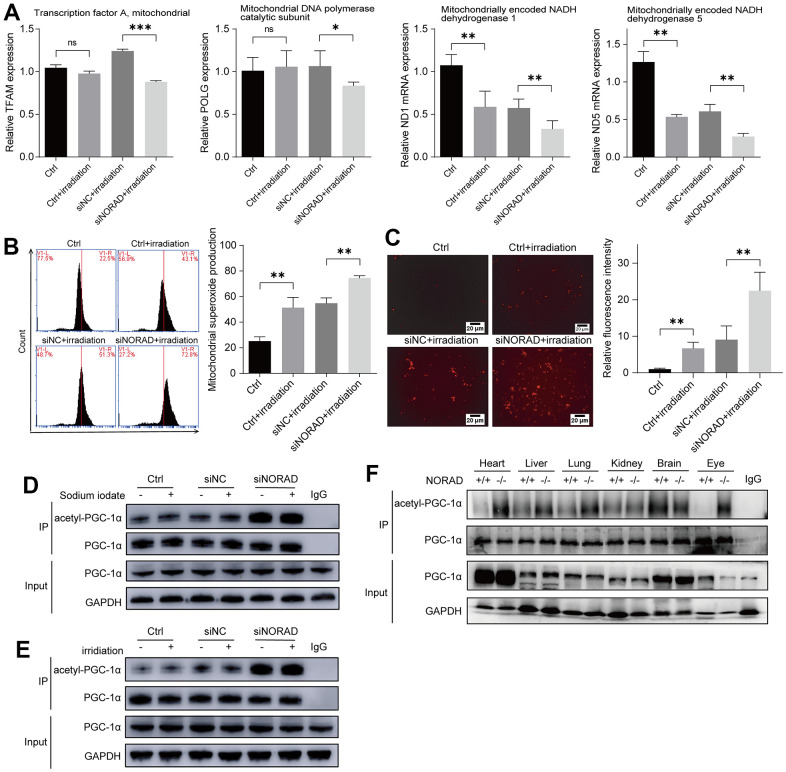
**NORAD knockdown increased the PGC-1α acetylation and mitochondrial ROS.** ARPE-19 cell lines were transfected with siRNA of siNORAD or siNC, after 96 hours, the cells were observed for mitochondrial damage, mitochondrial ROS, PGC-1α. (**A**) NORAD knockdown decreased the level of mRNA of mitochondrial transcription factor A (TFAM), mitochondrial DNA polymerase catalytic subunit (POLG), mitochondrially encoded NADH dehydrogenase 1 (ND1), and mitochondrially encoded NADH dehydrogenase 5 (ND5) in irradiation treated ARPE-19. (**B**) Flow cytometry was used to detect the mitochondrial ROS levels. Irradiation increased the mitochondrial ROS and NORAD knockdown aggravates irradiation-induced the rise of mitochondrial ROS. Data were from three separate experiments and described as mean ± SD. **P < 0.01. (**C**) NORAD knockdown aggravated irradiation-induced ROS production. The ROS levels were observed under an inverted fluorescence microscope. Images were representative of three separate experiments. n = 3, **P < 0.01. (40X, measure scar 20um) (**D**) Immunoprecipitation and western blot analysis of acetyl-PGC-1α levels in ARPE-19 transfected with siNORAD and exposed to sodium iodate. (**E**) Immunoprecipitation and western blot analysis of acetyl-PGC-1α levels in ARPE-19 transfected with siNORAD and exposed to irradiation. (**F**) Immunoprecipitation and western blot analysis of acetyl-PGC-1α levels in heart, liver, lung, kidney, brain, and eye of NORAD knockout mouse.

To verify the results of increased ocular mitochondrial ROS *in vivo*, we examined mitochondrial damage and mitochondrial ROS production *in vitro* using an irradiation-induced AMD model. To explore the effect of NORAD-knockdown on mitochondrial genes under irradiation, we conducted an RT-qPCR experiment and determined that irradiation decreased the mitochondrial homeostasis factors TFAM and POLG and mitochondrial respiratory chain complex genes ND1 and ND5 compared to a control group. However, NORAD-knockdown exacerbated the decrease of these mitochondrial genes (Figure 5A). MitoSOX staining revealed that irradiation increased the mitochondrial ROS compared with the control group. By contrast, the increase in mitochondrial ROS production was further aggravated upon NORAD-knockdown treatment (Figure 5B). The results were validated by fluorescence microscope photography (Figure 5C). As only non-acetylated PGC-1α can activate mitochondrial oxidative processes and inhibit the production of mitochondrial ROS, we examined PGC-1α acetylation under conditions of pathological damage. We found that NaIO_3_ and irradiation did not increase PGC-1α acetylation. Conversely, NORAD knockdown increased PGC-1α acetylation under both pathological and non-pathological conditions (Figures 5D, 5E). To validate the effect of NORAD on PGC-1α, we detected PGC-1α acetylation in various organs of NORAD-knockout mice. NORAD knockout increased PGC-1α acetylation in the heart, liver, lung, and eye, but was less explicit in the kidney and brain (Figure 5F). These findings indicate that the effect of NaIO_3_ and irradiation on PGC-1α acetylation is not obvious; nevertheless, NORAD knockdown could promote PGC-1α acetylation.

### NORAD knockdown promoted the binding of PGC-1α and SIRT1

**Figure 6:**
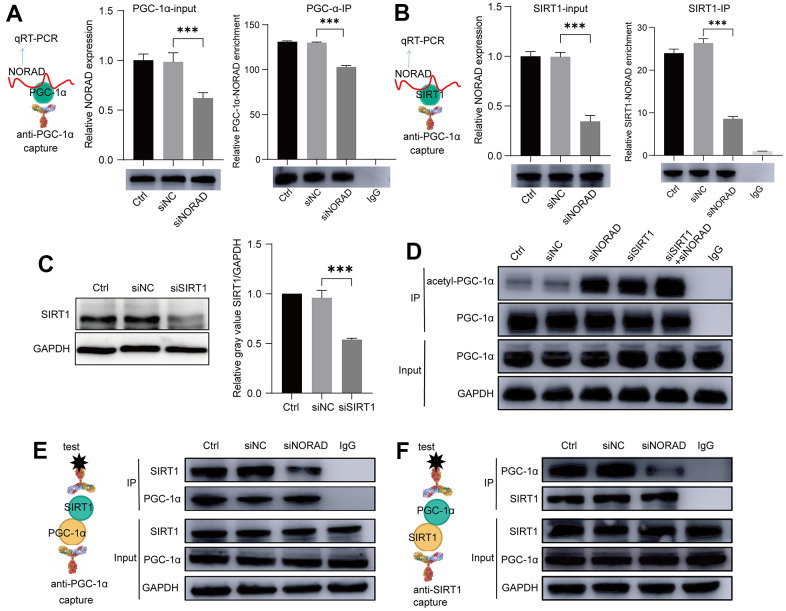
**NORAD knockdown increased the binding of PGC-1α and SIRT1.** ARPE-19 cell lines were transfected with siNORAD or siNC, after 96 hours, the interaction of NORAD with PGC-1α and SIRT1 was detected. (**A**) RIP assay confirmed the interaction of NORAD and PGC-1α. All groups of ARPE-9 were incubated with the anti-PGC-1α or anti-IgG antibody, and the precipitated complexes were analyzed with RT-qPCR to investigate the expression of NORAD. n = 3, ***P < 0.001. (**B**) RIP assay confirmed the interaction of NORAD and SIRT1. All groups of ARPE-9 were incubated with the anti-SIRT1 or anti-IgG antibody, and the precipitated complexes were analyzed with RT-qPCR to investigate the expression of NORAD. n = 3, ***P < 0.001. (**C**) ARPE-19 was transfected with siSIRT1 or normal contrast siNC. SIRT1 levels were analyzed through western blot. n = 3, ***P < 0.001 vs. siNC. (**D**) Immunoprecipitation and western blot analysis of acetyl-PGC-1α levels in ARPE-19 transfected with siNORAD, siSIRT1, or normal contrast siNC. (**E**) IP experiment confirmed the interaction of SIRT1 and PGC-1α. All groups of ARPE-9 were incubated with the anti-PGC-1α or anti-IgG antibody, and the precipitated complexes were analyzed with anti-SIRT1 by western blot. n = 3. (**F**) IP experiment confirmed the interaction of PGC-1α and SIRT1. All groups of ARPE-9 were incubated with the anti-SIRT1 or anti-IgG antibody, and the precipitated complexes were analyzed with anti-PGC-1α by western blot. n = 3.

According to the literature, PGC-1α, SIRT1, and NORAD interact with one another, so we performed RNA binding protein immunoprecipitation assay experiments (RIP) to determine whether both PGC-1α and SIRT1 interact with NORAD (Figures 6A, 6B). On the premise that the degree of binding between SIRT1 and PGC-1α influenced PGC-1α acetylation, we then detected PGC-1α acetylation after SIRT1 knockdown, NORAD knockdown, and combined SIRT1 and NORAD knockdown using an immunoprecipitation (IP) experiment. SIRT1 knockdown was confirmed by WB (Figure 6C). We verified that both SIRT1 knockdown and NORAD knockdown increased PGC-1α acetylation with similar effects (Figure 6D). The simultaneous knockdown of SIRT1 and NORAD had a cumulative effect on PGC-1α acetylation (Figure 6D). The presence of both SIRT1 and NORAD, therefore, exerts an effect on PGC-1α acetylation. We also hypothesized that the presence of NORAD facilitates SIRT1 and PGC-1α binding. Therefore, we detected SIRT 1 and PGC-1α binding through co-immunoprecipitation (co-IP) experiments. When we captured SIRT1 with antibodies against PGC-1α, we were able to drag SIRT1 down (Figure 6E); conversely, we were also able to drag down PGC-1α with antibodies against SIRT1 (Figure 6F). Thus, SIRT1 and PGC-1α were bound to each other. Surprisingly, when NORAD was knocked down, the amount of SIRT1 dragged down by the PGC-1α antibody and the amount of PGC-1α dragged down by the SIRT1 antibody both decreased (Figure 6E, 6F). These outcomes indicate that the presence of NORAD is conducive to the binding of SIRT1 and PGC-1α, thus promoting PGC-1α deacetylation by SIRT1.

## DISCUSSION

A specific dose of irradiation can cause retinal cells to produce AMD characterization that promoted aging and apoptosis and increased AMD-related gene makers. Using an AMD cell model, we investigated the effect of NORAD knockdown on retinal cells. We confirmed that NORAD-knockdown retinal cells were more susceptible to radiation damage, thereby generating higher AMD signs, and were more likely to age into AMD. The mitochondria of NORAD-knockdown retinal cells are also more vulnerable to radiation damage, causing the production of more mitochondrial ROS and activation of more p-P53 and P21, which, in turn, lead to the aging and apoptosis of retinal cells. We induced a mouse model of AMD with NaIO_3_ and found that the retina of the NORAD knockout mouse suffered more serious damage, consistent with the results of cell experiments. More importantly, NORAD-knockdown upregulated PGC-1α acetylation, thereby suggesting that NORAD may increase PGC-1α and SIRT1 interaction and promote the deacetylation of SIRT1 to PGC-1α. Furthermore, NORAD could interact with SIRT1 and PGC-1α. Together, these findings shed light on the molecular mechanism whereby NORAD-knockdown retinal cells may increase the risk of AMD and provide a potential target for AMD treatment.

AMD is a complex disease caused by a variety of environmental and genetic factors [42]. Many *in vitro* models of AMD have been established. As we know, the approaches of exposing A2E-laden ARPE-19 cells to blue light [36], the NaIO_3_-induced AMD cell model [35], and the dox-induced AMD cell model [32] all take advantage of a drug’s effect on cell damage, causing RPE cell death. Transmitochondrial cybrid cell lines that contain identical nuclei but possess mitochondria from AMD patients exploit the mitochondrial damage in the pathogenesis of AMD [43]. Given the aging-inducing properties of X-radiation [39], we irradiated the ARPE-19 cell line for some time to accelerate its senescence and eventually develop AMD-related markers. We irradiated the ARPE-19 cell line for some time to accelerate its senescence and eventually develop AMD-related markers. Our model is simple and close to the real environment of eye damage.

In the initial studies, NORAD was associated with genomic instability, and most subsequent research linked NORAD dysregulation to many cancers [18, 44]. Downregulation of NORAD promotes the production of apoptosis-related proteins by increasing PARP, caspase-3, and Bax and inhibits the proliferation and invasion of gastric cancer cells [45]. NORAD knockdown suppressed the proliferation and invasion of osteosarcoma cells because NORAD acted as a sponge for miR-199a-3p [46]. Next, removing NORAD drives premature aging in mice [22]. Our laboratory determined immediately that NORAD knockdown induces cell cycle arrest and aggravates ox-LDL-induced cell senescence of HUVECs, which promotes Ox-LDL-induced vascular endothelial cell injury and atherosclerosis [47]. Recently, the increased m6A modification level of NORAD reduced the sealing effect of PUMILIO protein, inhibited the expression of E2F3, and led to the aging of nucleus pulposus cells and intervertebral disc degeneration. These results all proved the significance of NORAD in aging. Similarly, we determined that NORAD knockdown accelerates the senescence of irradiation-induced ARPE-19 and exacerbates the AMD phenotype, thereby further proving the close relationship between NORAD and aging degenerative diseases. Therefore, NORAD may be a good target for the treatment of degenerative diseases.

Aging, sun exposure, low antioxidant intake, and other factors can cause mitochondrial damage that affects genes encoding the mitochondrial electron transport chain resulting in ROS overproduction [48]. Such changes will eventually lead to senescence and damage of RPE cells, resulting in retinal degeneration and AMD [48]. We confirmed that NORAD-knockdown damages the genes on the respiratory chain NADH1 and NADH5 and the mitochondria replication and transcription genes TFAM and POLG, which induce higher mitochondrial ROS production and a higher level of senescence-related factors p-P53 and P21. TFAM decreases ROS production by controlling the expression of mitochondrial ROS-generating enzymes, such as nicotinamide adenine dinucleotide phosphate oxidases [49]. In addition, considering that loss of PGC-1α or repression of the pathway of PGC-1α could induce impairment of RPE such as increasing mitochondrial ROS [50], promotion of retinal degeneration [51], metabolic dysfunction [52–54], and AMD-like phenotypes in mice [55, 56]. We further examined the PGC-1α level and PGC-1α acetylation activation. Interestingly, only the acetylated PGC-1α increased in NORAD-knockdown RPE while the total level of PGC-1α is unchangeable. As Rodgers had demonstrated that PGC-1α deacetylation is accomplished by an increased transcription level of SIRT1 [57], and, then we detected the level of SIRT1 after the NORAD knockdown, while the expression level of SIRT 1 is still unchangeable. Note that NORAD can inhibit PUM by the formation of phase-separated PUM condensates, thereby revealing a mechanism by which RNA-driven phase separation can regulate RNA-binding-protein activity and which may be a widely used mechanism of lncRNA-mediated regulation [19]. One prior work reported that LINC00842 binds to and prevents acetylated PGC-1α from deacetylation by deacetylase SIRT1 to form PGC-1α and consequently enhance the malignant phenotypes of PDAC cells [58]. Thus, we examined whether the absence of NORAD affected the binding of SIRT1 and PGC-1α using RIP and IP experiments. Surprisingly, we observed decreased binding between SIRT1 and PGC-1α after the NORAD knockdown. In contrast to LINC00842, NORAD promotes the deacetylation of PGC-1α by SIRT1 to activate PGC-1α. Our findings identify one mechanism by which lncRNA regulates the activity between proteins through binding proteins. Given the important role of PGC-1α in aging, NORAD and PGC-1α may also play an important role in other age-related degenerative diseases.

We acknowledge some limitations and confusion in our current study. Although we focused simply on the effect of NORAD knockdown in retinal cells on the formation and development of AMD with exposure to irradiation, the effect of NORAD overexpression on AMD is worth investigating. We tried to examine that effect but were unable to find a way to overexpress NORAD in retinal cells without impairing the state of the cell itself. In addition, better irradiation conditions and conditions for AMD formation need to be further explored. Irradiation mouse models of AMD are also unavailable, so we used NaIO_3_-induced AMD on mice. Finally, we found that after NORAD knockout, the PGC-1α acetylation level was increased in the eyes, heart, liver, and lungs, but ROS was decreased in the lung. No increased acetylation was observed in the kidney and brain. These results suggest that the role of NORAD in different organs may vary, along with different mechanisms involved.

**Figure 7:**
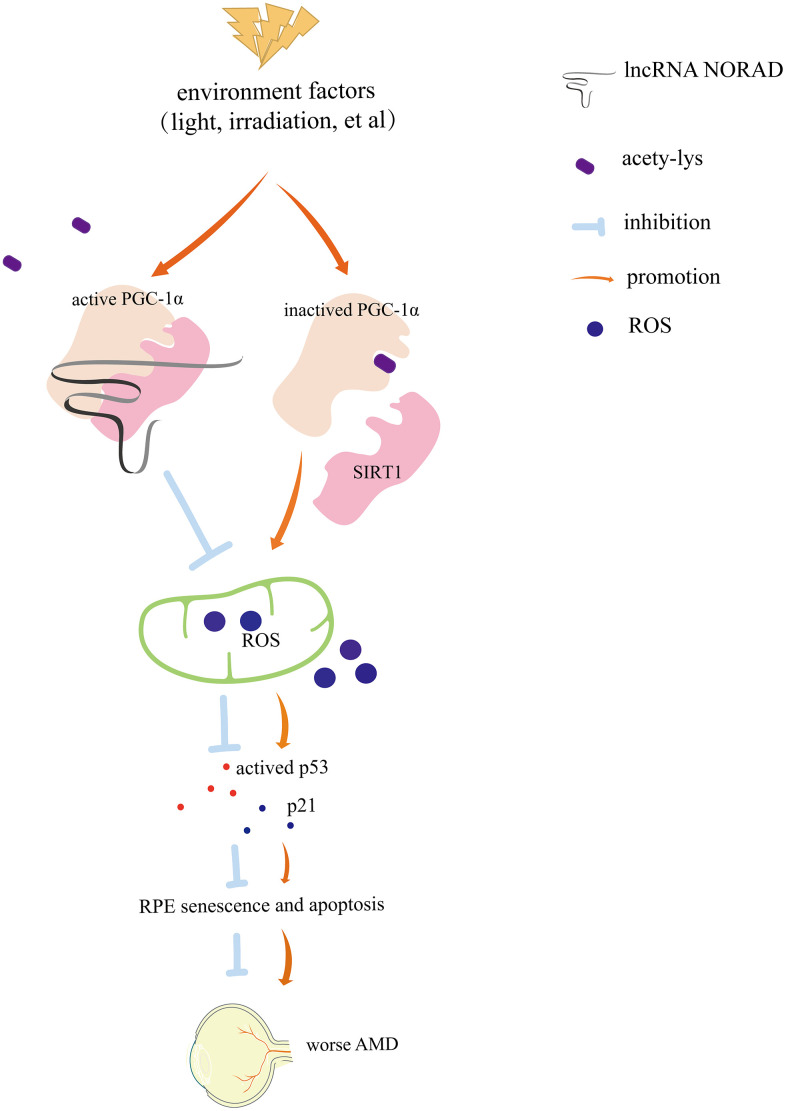
**Diagram of mechanism.** Environmental damage factors such as light exposure and irradiation could damage the retina and retinal pigment epithelial (RPE). NORAD-knockdown accelerates retinal cell senescence, apoptosis via PGC-1α acetylation, mitochondrial ROS, and the p-P53–P21signaling pathway, which resulting in worsening AMD. NORAD-mediated effect on PGC-1α acetylation might occur through the direct interaction with PGC-1α and SIRT1.

In summary, these results shed light on the important role of lncRNA NORAD in AMD development and progression (Figure 7) and provide a foundation for developing more targeted AMD therapies and predicting whether low NORAD expression is likely linked to the development of AMD or other diseases of aging.

## MATERIALS AND METHODS

### Cell culture

Adult retinal pigment epithelial cell line-19 (ARPE-19) (obtained from the ATCC) were routinely cultured in DME/F12 (HyClone, USA) containing 10% fetal bovine serum (BI) and 100 U penicillin/0.1 mg ml-1streptomycin at 37° C in an incubator with 5% CO2.

### Irradiation for AMD cell model

The cells were plated in six-well plates and irradiated. Before irradiation, the cell medium was changed into a serum-free medium. The total irradiation dose was 25 Gy and the dose rate was 100 cGy/min (X-ray 320, North Branford, Connecticut, Precision X-Ray). After irradiation, the cells were placed in an incubator with 5% CO2 for 6 h and replaced with 2 ml of complete medium. After 24 h of culture, SA-β-gal staining was carried out, and related markers of AMD were detected after 96 h.

### RNA extraction and RT-qPCR analysis

Total RNA was extracted from cultivated cells using Trizol reagent (Takara, Shiga, Japan). Approximately 1 mL of Trizol was directly added to each sample. Then, 200 ul of chloroform was added to each sample after 30 min of ice cracking, eddy shock of 15–30s, and suspension for 15 min on ice. The sample was subsequently centrifuged at 1200 rpm/min at 4° C for 15 min, 400 ul of the topmost phase was absorbed, 500 ul of isopropyl alcohol was added and mixed gently, and the solution was allowed to stand on ice for 10 min. After centrifugation at 1000 rpm/min at 4° C for 10 min, RNA precipitation was observed. The cDNAs were synthesized using the PrimeScript™ RT reagent Kit with gDNA Eraser (Takara, Shiga, Japan). Quantitative RT-qPCR was performed with the StepOnePlus Real-Time PCR System (Thermo Fisher Scientific, USA). The CT value was obtained as follows: ∆Ct = CT (target gene) – CT (reference gene), ∆∆Ct = ∆Ct (treatment group) − ∆Ct (control group). The relative expression of the target genes was calculated using 2−∆∆Ct.

### Enzyme-linked immunosorbent assay (ELISA) in culture supernatants

Total supernatant was collected from irradiated-ARPE-19 groups or siRNA transfected groups cultured in six-well plates after 96 h at the last medium change (2 ml for every well). ELISA kits (see Supplementary Table 2) were used to measure the secretion of VEGF-A (dilution of supernatant 1:20), complement component 3 (dilution of supernatant 1:20), Aβ-40, Aβ-42 (dilution of supernatant 1:15) in the culture supernatants. Note that dilutions were performed to ensure that the protein of interest was within the concentration range for detection and quantification. All samples were tested in duplicates following the manufacturer’s instructions. Immediately after adding the stop solution, optical densities/absorbance readings were obtained at 450 nm using a plate reader (INFINITE M NANO, TECAN). The average of the duplicate readings was used to estimate the concentration using the standard curve.

### Cell apoptosis assay

ARPE-19 was seeded on six-well plates at a cell density of 1.5 × 10^5^ cells/well. After siRNA transfection for 24 h, the cells were irradiated. After 72 h, cells of all groups were collected and suspended with 100μL of 1x binding buffer. Then, 5uL of PI and 5uL of Annexin V-FITC solutions (MAO220, Meilun, China) were added to the cells. After incubation for 15 min, 400 μL of binding buffer was supplemented into the cells. The apoptosis rate of cells was assessed using a flow cytometer (BD Accuri® C6 Plus, USA).

### Cell cycle assay

The ARPE-19 were seeded on six-well plates and cultured for 24 h. After siRNA transfection for 24 h, the cells were irradiated. A cell cycle detection kit (MAO334, Milunbio, China) was used to measure cell cycle distribution after 72 h. Briefly, the cells were fixed with 70% ethanol overnight at 4° C, washed once by PBS, diluted with the pre-configured propidium iodide/RNase staining solution, and incubated for 30 min at room temperature protected from light. Cell cycle distribution was detected and analyzed with a flow cytometer (BD Accuri® C6 Plus, USA).

### Determination of ROS

Mitochondrial superoxide was detected using the fluorescent MitoSOX probe (Invitrogen, USA). A 5μM MitoSOX™ reagent working solution was prepared. Six hours after irradiation, all cell samples were collected into 1.5 ml EP tubes. Then, 1.0 ml reagent working solution was added to the resuspended cells in EP tubes. The cells were incubated for 10 min at 37° C while protected from light. The cells were gently washed thrice with PBS. Finally, the cells were assessed using a flow cytometer (BD Accuri® C6 Plus, USA).

### Co-immunoprecipitation and PGC-1α acetylation level detection

Using two tubes of ARPE-19 protein supernatant (1.8 mL/tube), 10μL of anti-PGC-1α antibody was added to one tube and IgG to the other tube as the negative control. The mixture was kept at 4° C for 2 h, then 50 μ L of protein A/G (Millipore, USA) was added to both tubes. The tubes continued cooling overnight at 4° C. The precipitates were collected by centrifugation for 1 min, washed with washing buffer I (see Supplementary Table 3) 4 times (1 mL/time), and then washed with 1 mL of washing buffer II (see Supplementary Table 3) once. The obtained precipitates were immunoprecipitated complexes. An appropriate amount of protein loading buffer was added to the precipitate complex and the precipitates were kept at 95° C for 5 min. After centrifugation, the supernatant was taken for the BCA experiment (BCA Protein Assay Kit, Beyotime, China) and for Western blot. SIRT1 antibody (Protintech, China) was used to detect the bond between SIRT1 and PGC-1α. Acetylated-lysine antibody was employed to test the level of PGC-1α acetylation. Experimental groups will vary according to different experimental designs.

### RNA-binding protein immunoprecipitation (RIP) assay

A RIP kit (GENESEED, China) was used to perform the RIP assay. In brief, the cell lysates were respectively added to a magnetic bead conjugated with a human anti-PGC-1α, anti-SIRT1 antibody, and control IgG in RIP buffer. The immunoprecipitation was digested with proteinase K to purify the immunoprecipitated RNA. qRT-PCR was performed to verify the presence of NORAD.

### Western blot

The ARPE-19 in each group of animal organ tissue was lysed using RIPA lysis buffer (Solarbio Biotechnology, Beijing, China). Equal amounts of proteins were separated with SDS-polyacrylamide gels and then transblotted onto a PVDF membrane. The membranes were incubated with 5% dried nonfat milk buffer for 2 h to prevent nonspecific binding. They were then incubated with the relevant primary antibodies and with a secondary antibody. The protein bands in the membranes were detected by enhanced chemiluminescence detection reagents (Biosharp, Anhui, China) and analyzed with Image J.

### RNA interference

The siNORAD, siSIRT1, and negative siNC were from GenePharma Co., Ltd. (Shanghai, China). The siNORAD sequence consisted of the sense strand GCUGUCGGAAGAGAGAAAUTT and the antisense strand AUUUCUCUCUUCCGACAGCTT. The sequence of siNC consisted of the sense strand UUCUCCGAACGUGUCACGUTT and the antisense strand ACGUGACACGUUCGGAGAATT. The ARPE-19 were transfected with siRNAs by using HiPerFect (Qiagen, China). For irradiation treatment, the cells were exposed to a dose of irradiation after transfection for 24 h. SIRT1 mRNA was knocked down in the same way described above. The SIRT1 siRNA of the sense strand is CGGGAAUCCAAAGGAUAAUT and the antisense strand is AUUAUCCUUUGGAUUCCCGTT.

### OCT, funduscopic examination, and electroretinography

Six-week-old male NORAD−/− mice were purchased from BIOCYTOGEN (Beijing, China) and six-week-old male C57BL/6 J mice were purchased from Pengyue (Jinan, China). Three NORAD knockout mice and three wild-type mice were randomly selected to receive tail vein injections of NaIO_3_ (Sigma-Aldrich, China) (30mg/kg). Testing began seven days after the injection of NaIO_3_. OCT and funduscopic examination (REMEGEN, Yantai, China) were performed on day 7. Electroretinography was performed on day 8.

### Mouse organ mitochondrial ROS experiments and mouse eye AMD marker detection

At the later stage, three 12-month-old male NORAD−/− mice and six 12-month-old male wild-type mice were randomly selected. Three NORAD−/− mice and three wild-type mice received tail vein injections of NaIO_3_ (Sigma-Aldrich, China) (30mg/kg). Eight days later, the heart, liver, lungs, kidneys, brains, and eyes (one eye is used for RT-qPCR experiments later) were dissected from mice. All organs were filtered into a single-cell solution by a cell strainer (Biosharp, China), then washed five times with PBS for the determination of the ROS experiment. The remaining eyes were employed for the detection of AMD markers such as C3, ICAM-1, APP, APOE, and CRYAA (for the primer sequence see Supplementary Table 1).

### Mouse organ PGC-1α acetylation level detection

Three 12-month-old female NORAD−/− mice and three wild-type mice were randomly selected to obtain a single-cell solution of heart, liver, lungs, kidneys, brains, and eyes; then, all samples were used to detect the level of PGC-1α acetylation (using the same operations described above).

### Statistical analysis

Results were processed using SPSS. The statistical charts were made by GraphPad prism 8. Data were shown as mean values ± standard deviation (SD). T-test was used to analyze the data differences, and P < 0.05 indicated statistically significant differences.

## Supplementary Material

Supplementary Tables
