# Supplementary material for: LncRNA NORAD defects deteriorate the formation of age-related macular degeneration
Source: Aging (Albany NY). 2023 Jul 29;15(15):7513–32. doi: 10.18632/aging.204917 (PMC10457045; doi:10.18632/aging.204917)
Supplement: Supplementary Tables [file aging-15-204917-s001.pdf]

SUPPLEMENTARY TABLES

Supplementary Table 1. qRT-PCR primer sequence.

Name	Forward primer (5'-3')	Reverse primer (5'-3')
VEGF-A(Human)	TCTTCAAGCCATCCTGTGTG	ATCCGCATAATCTGCATGGT
APP(Human)	CCGCTGCTTAGTTGGTGAGTTTGT	ACGGTGTGCCAGTGAAGATGAGTT
APOE(Human)	AACTGGCACTGGGTCGCTTT	GCCTTCAACTCCTTCATGGTCTCGT
C3(Human)	GGGGAGTCCCATGTACTCTATC	GGAAGTCGTGGACAGTAACAG
ICAM- 1(Human)	ATGCCCAGACATCTGTGTCC	GGGGTCTCTATGCCCAACAA
NORAD (Human)	TGATAGGATACATCTTGGACATGGA	AACCTAATGAACAAGTCCTGACATACA
GAPDH (Human)	GGAGCGAGATCCCTCCAAAAT	GGCTGTTGTCATACTTCTCATGG
TFAM (Human)	ATGGCGTTTCTCCGAAGCAT	TCCGCCCTATAAGCATCTTGA
PLOG (Human)	GCTGCACGAGCAAATCTTCG	GTCCAGGTTGTCCCCGTAGA
ND1(Human)	TTCTAATCGCAATGGCATTCCT	AAGGGTTGTAGTAGCCCGTAG
ND5 (Human)	TTCATCCCTGTAGCATTGTTCG	GTTGGAATAGGTTGTTAGCGGTA
APP (Mouse)	TCCGAGAGGTGTGCTCTGAA	CCACATCCGCCGTAAAAGAATG
APOE (Mouse)	CTGACAGGATGCCTAGCCG	CGCAGGTAATCCCAGAAGC
CRYAA (Mouse)	ACGGCAAACACAACGAGAGG	CATGCCATCAGCAGACAGG
C3(Mouse)	CCAGCTCCCCATTAGCTCTG	GCACTTGCCTCTTTAGGAAGTC
ICAM- 1 (Mouse)	GTGATGCTCAGGTATCCATCCA	CACAGTTCTCAAAGCACAGCG
GAPDH (Mouse)	AGGTCGGTGTGAACGGATTTG	TGTAGACCATGTAGTTGAGGTCA
NORAD (Mouse)	CGCTGTCGCCGTAGAAGTCC	GACGAGTGTCGCTCCTGGGT

Supplementary Table 2. The origin of reagent and resource.

Regent	Manufacturers	Identifier
Aβ-40 ELISA	Thermo Fisher	KHB3481
Aβ-42 ELISA	Thermo Fisher	KHB3441
VEGF-A ELISA	Invitrogen	KHGO111
Complement component 3 (C3) ELISA	Abacam	ab108823
MitoSOXTM Red mitochondrial superoxide indicator	Invitrogen	M36008
RNA Immunoprecipitation Kit	GENESEED	P0101
Protein G Plus/Protein A Agarose Suspension	Millipore	IP05-1.5ml
ECL Chemiluminescence substrate (ultra-sensitive)	Biosharp	BL520A
HiPerFect Transfection Reagent	QIANGEN	301705
Sodium iodate	Sigma	S4007
Cell Cycle and Apoptosis Analysis Kit	Milunbio	MAO334
Annexin V-FITC/PI Apoptosis detection Kit	Milunbio	MAO220
p21 Rabbit Polyclonal Antibodies	Proteintech	10355-1-AP
Phospho-P53 (Ser15) Rabbit Recombinant	Proteintech	80195-1-RR
SIRT1 Rabbit Polyclonal antibody	Proteintech	13161-1-AP
PGC-1α Mouse Monoclonal antibody	Proteintech	66369-1-lg
Acetylated-lysine Antibody	Cell Signaling	9441
GAPDH Rabbit mAb	Cell Signaling	D16H11
Goat anti-Mouse IgG (H+L) Secondary Antibody	ZSGB-BIO	ZB-2305
Goat anti-Rabbit IgG (H+L) Secondary Antibody	ZSGB-BIO	ZB-2301

Supplementary Table 3. IP experiment wash buffer preparation.

Wash buffer I	Final concentration	Total volume 100ml
HEPEs	10mmol/L	1ml
NaCl	100nmol/L	10ml
EDTA	5nmol/L	1ml
Tween20	0.10%	0.5ml
Benzamidine	10mmol/L	0.016g
BSA	3%	3g
ddH2O		to 100 ml
**Wash buffer II**	**Final concentration**	**Total volume 100ml**
HEPEs	10mmol/L	1ml
NaCl	100nmol/L	10ml
EDTA	5nmol/L	1ml
Tween20	0.10%	0.5ml
Benzamidine	10mmol/L	0.016g
ddH2O		to 100 ml
